# The handedness-associated *PCSK6* locus spans an intronic promoter regulating novel transcripts

**DOI:** 10.1093/hmg/ddw047

**Published:** 2016-02-21

**Authors:** Robert Shore, Laura Covill, Kerry A. Pettigrew, William M. Brandler, Rebeca Diaz, Yiwang Xu, Javier A. Tello, Joel B. Talcott, Dianne F. Newbury, John Stein, Anthony P. Monaco, Silvia Paracchini

**Affiliations:** ^1^School of Medicine, University of St Andrews, St Andrews KY169TF, UK,; ^2^Wellcome Trust Centre for Human Genetics, University of Oxford, Oxford OX3 7BN, UK,; ^3^School of Life and Health Sciences, Aston University, Birmingham, UK and; ^4^Department of Physiology, Anatomy & Genetics, Parks Rd., Oxford OX1 3PT, UK

## Abstract

We recently reported the association of the *PCSK6* gene with handedness through a quantitative genome-wide association study (GWAS; *P* < 0.5 × 10^−8^) for a relative hand skill measure in individuals with dyslexia. PCSK6 activates Nodal, a morphogen involved in regulating left–right body axis determination. Therefore, the GWAS data suggest that the biology underlying the patterning of structural asymmetries may also contribute to behavioural laterality, e.g. handedness. The association is further supported by an independent study reporting a variable number tandem repeat (VNTR) within the same *PCSK6* locus to be associated with degree of handedness in a general population cohort. Here, we have conducted a functional analysis of the *PCSK6* locus combining further genetic analysis, *in silico* predictions and molecular assays. We have shown that the previous GWAS signal was not tagging a VNTR effect, suggesting that the two markers have independent effects. We demonstrated experimentally that one of the top GWAS-associated markers, rs11855145, directly alters the binding site for a nuclear factor. Furthermore, we have shown that the predicted regulatory region adjacent to rs11855415 acts as a bidirectional promoter controlling the expression of novel RNA transcripts. These include both an antisense long non-coding RNA (lncRNA) and a short *PCSK6* isoform predicted to be coding. This is the first molecular characterization of a handedness-associated locus that supports the role of common variants in non-coding sequences in influencing complex phenotypes through gene expression regulation.

## Introduction

Handedness is one of the most researched human behavioural traits, yet one of the least understood. The vast majority of people worldwide prefer using their right hand for writing and performing most tasks. This observation implies a disadvantage for left-handedness and a large body of research has investigated whether being left-handed can increase susceptibility to particular traits or disorders (1). In this context, language-related disorders have been a particular focus of attention due to handedness showing a correlation, albeit weak, with language dominance lateralization (2). However, no robust evidence supports the association of handedness with disease risk.

Handedness presents a weak but very consistent heritability across different studies, estimated to be ∼25% (3). The assessment of hand preference is relatively trivial and has been collected for tens of thousands of individuals for which genome-wide genotype data are available. However, no gene or variant has yet been identified to be associated with a left-handed status, suggesting a complex and multifactorial origin of this trait (4). Recently, we have identified *PCSK6* (proprotein convertase subtilisin/kexin type 6) as the first gene associated with a handedness-related measure at a statistically significant level (*P* < 0.5 × 10^−8^) in two separate studies (5,6). We conducted a genome-wide association study (GWAS) using a quantitative measure of relative hand skill [i.e. PegQ, derived from peg-board scores (7)]. *PCSK6* is known to control the activation of the Nodal pathway required for left–right axis determination during early embryonic development (8). Interestingly, the association appeared to be specific to individuals with dyslexia (*n* = 728) and did not replicate in an epidemiological cohort representing the general population (*n* = 2666) (6). The top associated marker in the initial discovery sample (*n* = 197) was rs11855415 (5), and the strongest associated marker in the most recent and larger GWAS was rs7182874 (6). While *PCSK6* was the only gene that reached statistical significance, other genes and pathways involved in the determination of left–right structural asymmetries showed association with handedness both in the cohort selected for dyslexia and in the epidemiological cohort. For example, the top associated gene in the general population cohort was located in proximity of *GPC3* (*P* = 2.08×10^−6^) (6), a gene implicated in cardiac laterality defects (9). These data suggest that the same biological pathways controlling structural laterality may be partly implicated in contributing to behavioural laterality.

**Figure 1.**
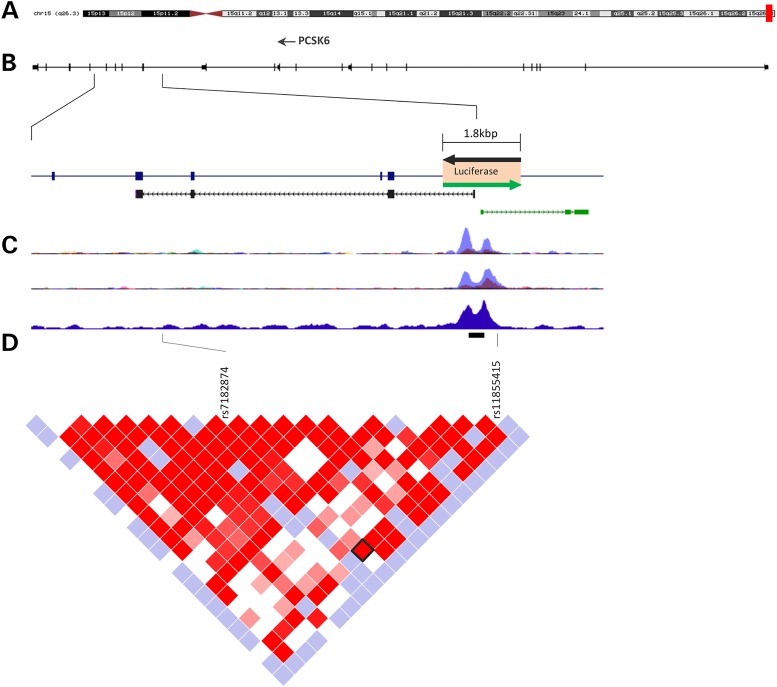
*PCSK6* locus associated with relative hand skills. (**A**) *PCSK6* is located on chromosome 15q26.3 as indicated by the red box. (**B**) *PCSK6* structure and expanded view of the region where top associations with relative hand skill cluster (5,6). Both sense (black) and antisense (green) transcripts are predicted to originate within this region, as shown by the human spliced EST and GENCODE tracks from the UCSC Genome Browser (http://genome-euro.ucsc.edu), respectively. A 1.8 kb region (orange box) was cloned into luciferase reporter vectors in both directions (black and green arrows). (**C**) This region is predicted to contain regulatory elements as shown by UCSC tracks for Chip-SEQ ENCODE (10). From top, the tracks show H3K27Ac and H3K4Me3 histone marks in different cell lines where the highest peak corresponds to the K562 cell line. RNA polymerase II binding for the K562 cell is shown in the third track. (**D**) The genetic associations cluster within a 12.7 kb LD region, visualized as D′ values, in HapMap CEPH data. The black bar indicates the position of the rs10523972 VNTR. Rs7182874, the strongest associated marker in the most recent GWAS (6), is in high LD (black diamond) with rs11855415, which was the top associated marker in the discovery sample (5) and shows functional allelic effects in the present study. LD visualization of the surrounding region is shown in Supplementary Material, Figure S1.

The single-nucleotide polymorphisms (SNPs) at the *PCSK6* locus that showed the highest association (6) lie close to an intronic region predicted to have a regulatory function (10) (Fig. 1 and Supplementary Material, Fig. S1). Both short-sense mRNA and antisense long non-coding RNA (lncRNA) molecules are predicted to be regulated by this region (Fig. 1B). The same locus has been found to be associated with degree of handedness, assessed by the Edinburgh questionnaire (11), in an independent sample representative of a general German population (12). This study did not find association with the rs11855415 marker [selected for replication of the first GWAS results (5)] but reported association with a 33 bp variable number tandem repeat (VNTR) rs10523972 (Fig. 1D). VNTRs have been shown to modulate gene expression and contribute to human diseases by directly affecting transcriptional regulation (13–16). It is therefore possible that the association with handedness observed for SNPs at the *PCSK6* locus (6) detected the VNTR effect.

In this study we investigate the molecular mechanisms underlying the association between *PCSK6* and handedness. Our genetic analysis and allele-specific assays support a functional role for the rs11855415 marker, but not the VNTR. Functional analysis of this locus in a range of cell lines has characterized a bidirectional promoter and the transcripts it regulates. These include novel molecules such as a short coding *PCSK6* isoform and alternatively spliced lncRNAs.

## Results

### Association analysis

We recently identified the first statistically significant association with a handedness-related measure (PegQ) at the *PCSK6* locus by screening cohorts of children selected for dyslexia (5,6). Top associations clustered in a region of high linkage disequilibrium (LD; Fig. 1D; Supplementary Material, Fig. S1). An independent study reported an association between degree of handedness and the VNTR rs10523972 at the same locus (12). The VNTR was not assessed in our previous GWASs (5,6). To assess whether the GWAS results were mediated by the VNTR, we conducted an association analysis in the same discovery sample of individuals with dyslexia where the *PCSK6* signal was originally detected (5). The strongest associated marker in this sample was rs11855415, which was imputed. Direct genotypes for both the VNTR and rs11855415 were available for 162 individuals from the discovery samples (*n* = 197). The VNTR allelic frequencies were similar to those previously reported by Arning *et al*. (12) with the most frequent alleles being the 6 (20%), the 9 (71%) and the 10 (6%) 33 base repeats. The two markers are not in LD (*r*^2^ = 0.05). We found no association for the VNTR, while the rs11855415 association with PegQ was confirmed in this subgroup (*P* = 4.8 × 10^−4^; Supplementary Material, Table S1). Differences in association *P*-values compared with the GWASs (5,6) are most likely due to the smaller sample size analysed here as a result of limited DNA availability. The concordance rate between imputed and directly genotyped data for rs11855415 was 98%. The VNTR genotypes were generated for 188 individuals from the GWAS discovery sample; no VNTR association was detected in this larger group either. VNTR alleles were analysed both individually as well as grouped into ‘short’ (<9 repeats) and ‘long’ (≥9 repeats), consistent with the analysis conducted by Arning *et al*. (12). In summary, by running association analysis in exactly the same individuals, our data rule out the possibility that the SNP associations previously reported (5,6) detected a functional effect of the rs10523972 VNTR.

### Allelic effect on transcription factor affinity

The top associations with PegQ (5,6) clustered within a 12.7 kb intragenic region of high LD (Fig. 1D). All 22 SNPs from the HapMap data [which included the top GWAS associations for PegQ (6)] within this LD region were tested for allelic effects on the loss/gain of transcription factor bind sites (TFBSs) using the TRANSFAC (17) and Genomatix MatInspector (18) databases (Supplementary Material, Table S2). The rs11855415 marker itself was predicted to affect the largest number of TFBSs. Interestingly, the allelic effect of rs11855415 is predicted to affect binding for several HOX genes (i.e. *HOXA5*, *HOXB3*, *HOXB8* and *HOXD10*) known to control the anterior–posterior developmental patterning of the body axis (19). The rs11855415 marker is located in close proximity to a region predicted to act as an intronic promoter (Fig. 1C) (20).

**Figure 2.**
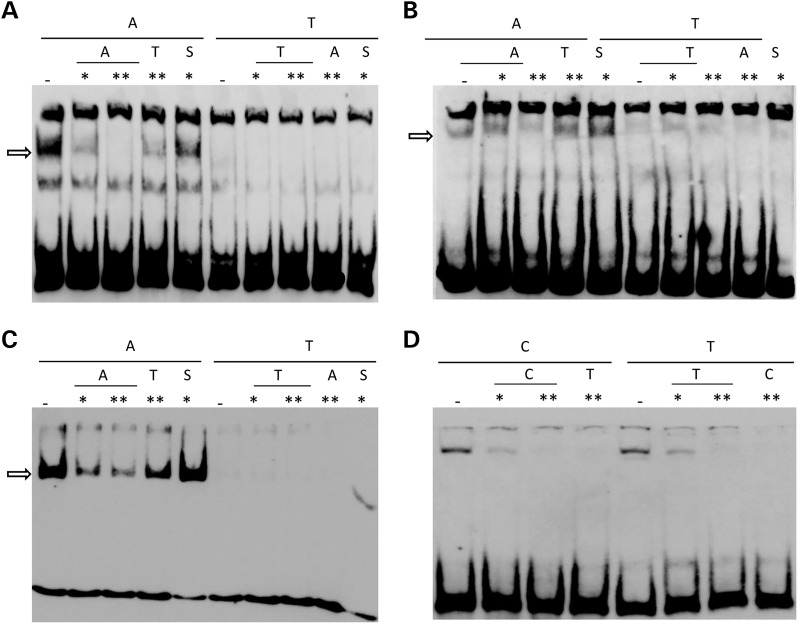
EMSA results. Probes centred on rs11855415 A and T alleles are incubated with nuclear extracts from (**A**) hNSC, (**B**) SH-SY5Y and (**C**) K562 cell lines. The arrow indicates a band appearing for allele A and absent for allele T, demonstrating DNA–protein binding. (**D**) No difference is observed between probes for rs7182874 C and T alleles when incubated with SH-SY5Y nuclear extracts. To test for specificity of the signal, different competitors where used as denoted for each lane: dash indicates no competitor; *(10-fold) and **(100-fold) indicate excess of cold competitor specified by the SNP alleles (specificity is shown by reduced signal for same allele as labelled probe while the opposite allele does not affect the binding); S is the scrambled competitor.

To confirm these *in silico* predictions, rs11855415 was tested *in vitro* for its effect on TFBS affinity using an Electrophoretic Mobility Shift Assays (EMSA). A clear difference in DNA–protein binding was consistently observed for the two rs11855415 alleles across a range of cell types, including neuronal cell lines (Fig. 2A–C). Specifically, rs11855415 the probe for allele A [minor allele; associated with relatively stronger right-hand abilities (5)] indicates bindings of transcription factors which are absent for the T allele probe. We also analysed rs7182874, which was the top associated marker in the most recent GWAS (6) and was also predicted to affect the binding sites of different transcription factors (Supplementary Material, Table S2). We observed no allelic differences in recruiting transcription factors (Fig. 2D). Rs7182874 is in strong LD with rs11855415 (*D*′ = 1; *r*^2^ = 0.62; Fig. 1D).

### Promoter characterization

**Figure 3.**
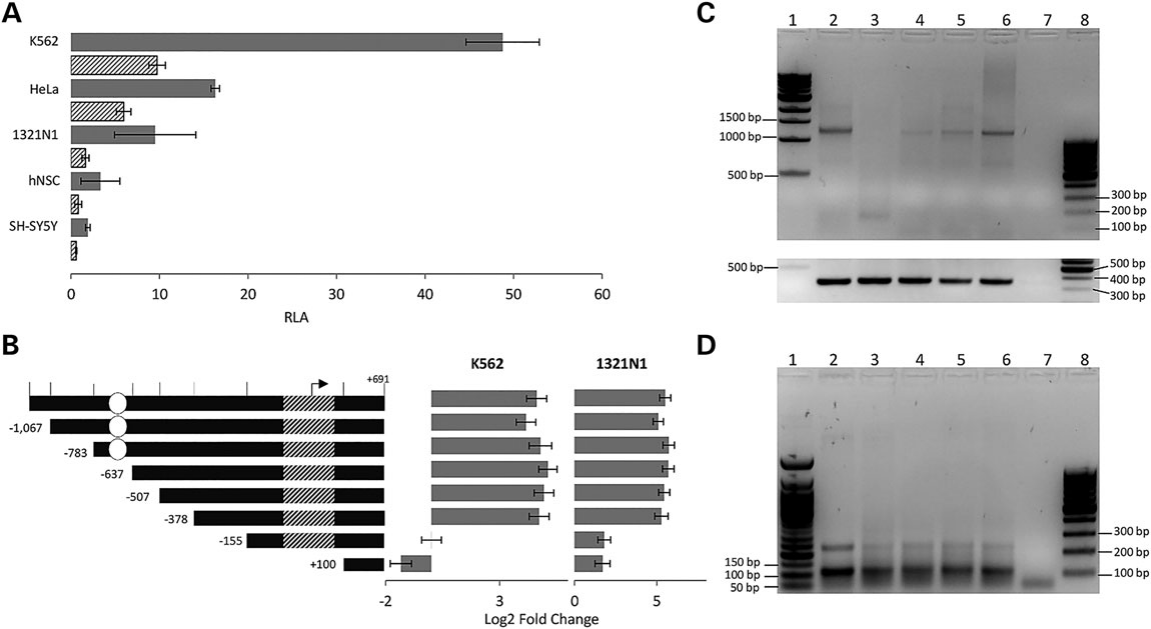
Characterization of the *PCSK6* region associated with handedness. (**A**) The genomic region spanning the regulatory elements at the *PCSK6* locus (orange box, Fig. 1B) was cloned into a luciferase reporter vector in both sense and antisense directions. Bars (grey for sense and striped for antisense direction) show mean FC in relative luciferase activity (RLA) following normalization with the Renilla construct and relative to the empty pGL4 vector in five cell lines. Bars indicate mean ± SD (*n* = 4). (**B**) Minimal promoter analysis of the sense construct. Luciferase-expressing constructs containing different segments of the region upstream of the TSS of the short-sense *PCSK6* isoform were analysed for promoter activity in K562 and 1321N1 cells. The rs11855415 SNP and the VNTR location are indicated by a white circle and a striped bar, respectively. An arrow indicates the position of the TSS of the *PCSK6* short-sense isoform. The vertical bars on the top segment indicates the sites targeted by restriction enzymes to generate the different constructs (from left to right: *Kpn*I, *Nde*i, *Awr*II, *San*Di, *Stu*I, *PfI*MI, *AcI*I and *Bsa*AI). Luciferase expression was measured relative to the empty pGL4 vector and log-transformed; all assays were performed in triplicate and repeated at least three times. Bars represent mean ± SD (*n* = 3). (**C**) Detection of a novel *PCSK6* isoform. A PCR fragment of 1247 bp spans a novel exon, immediately adjacent to the regulatory region tested in A) (see also Fig. 1) to the 3′ UTR of the full-length transcript (see Supplementary Material, Fig. S3). cDNA quality was confirmed for all cell lines by targeting the β-actin transcript (353 bp), a house-keeping gene (see Supplementary Material, Fig. S3). (**D**) A PCR fragment of 112 bp detecting the RNA antisense molecule PCSK6-AS1 with primers targeting the first and second exons. A less intense band of 232 bp corresponds to an alternatively spliced isoform (PCSK6-AS2) (see Supplementary Material, Fig. S3 for details of primer design). Sample order in both (C) and (D): lanes 2–6: K562, HeLa, 1321N1, hNSC and SH-SY5Y; lane 7: negative control; lanes 1 and 8, reference ladders (1 kb and 100 bp in C, and 50 bp and 100 bp in D).

The rs11855415 marker is located close to a predicted intronic regulatory region (Fig. 1D). This region is predicted to be a bidirectional promoter regulating both sense and antisense RNA molecules (Fig. 1B). To test for putative promoter activity, we cloned this region into luciferase gene reporter constructs in both sense and antisense directions (Fig. 3A). The cloned fragment was 1839 bp long in order to span the predicted promoter region and nearby genetic markers associated with handedness, including rs11855415 and the VNTR. Experiments were conducted across neuronal (1321N1, hNSC and SH-SY5Y) and non-neuronal (K562 and HeLa) cell lines selected for different criteria. Neuronal cells were chosen due to the high expression of *PCSK6* in the central nervous system (21,22). K562 showed the strongest signals for predicted promoter activity in the ENCODE tracks (Fig. 1C and Supplementary Material, Fig. S2) (10) at this locus. HeLa is a commonly used cellular model and is easily transfected.

Analysis of luciferase expression showed strongest promoter activity in the sense direction in all cell lines (Fig. 3A). In agreement with the ENCODE data (10) (Fig. 1C), the strongest activity of the reporter construct was detected in K562 cells. The rs11855415 marker is upstream of the predicted core promoter in the sense direction. To evaluate the potential effect of this SNP and further characterise this intronic promoter, we generated a series of progressive deletions from the 1839 bp long sense sequence (Fig. 3B). Analysis was conducted in the K562 cell line, as it displayed the strongest signal of promoter activity in both our experiments (Fig. 3A) and in the ENCODE data (10), and in the 1321N1 cell line, which provided the relatively strongest signal of promoter activity in the neuronal-type cells (Fig. 3A). The analysis revealed a minimal core promoter located in the region up to −155 bp relative to the transcription start site (TSS) of the shorter *PCSK6* isoform in both cell lines (Fig. 3B). The region spanning rs11855415 did not affect promoter activity. As expected, the VNTR marker sits within the core promoter. A consistent luciferase expression pattern was observed across the two cell lines.

We then assessed for the presence of transcripts predicted to be regulated by this intronic promoter. A sense RNA transcript was predicted by the human spliced expressed sequence tag (EST) catalogue from the UCSC Genome Browser (23) to have four exons (DB023826.1), the first of which is not included in the full-length or most common *PCSK6* isoforms (Fig. 1B). Contrary to this prediction (Fig. 1B), we were able to obtain PCR amplification of a transcript that fully extends to the 3′UTR of *PCSK6* using a primer targeting this predicted first exon (Fig. 3C; Supplementary Material, Fig. S3). A band with particular high intensity was observed in the K562 cell line, consistent with the promoter activity previously observed for this cell line (Figs 1C and 3A). A strong band was also observed for the neuronal-type SH-SY5Y cells, which did not display particularly strong activity in the luciferase assay (Fig. 3A). This inconsistency could be the result of low transfection efficiency of the promoter construct in the SH-SY5Y cells. A band was not detected in HeLa cells for which good quality of input RNA was confirmed, both by the detection of a house-keeping gene (Supplementary Material, Fig. S3) and detection of the antisense RNA (Fig. 3D). This short transcript is predicted to be coding and to maintain the domain downstream of the convertase site of the full-length PCSK6 protein (Supplementary Material, Fig. S4). We also identified novel alternatively spliced isoforms of the predicted lncRNA (*PCSK6-AS1* and *PCSK6-AS2)* in all cell lines (Fig. 3D). Sanger sequencing of PCR products confirmed the splicing predictions of both the shorter sense and the antisense RNA molecules.

Dual-luciferase gene reporter assays were used to assess allelic effects on promoter activity. We tested all possible VNTR /rs11855415 allele combinations, including those not available from human genomic DNA, which we generated using site-directed mutagenesis. We conducted the luciferase assays in the same cell lines used for the promoter characterisation; K562 and 1321N1. Consistent with the promoter characterisation experiments, we observed a higher signal in the K562 cells and stronger promoter activity in the sense direction compared with the antisense direction in both cell lines. No substantial allelic difference was observed for either genetic variant (Supplementary Material, Fig. S5). The only test that showed a suggestive allelic difference was for the 10A versus 10T haplotypes comparison in the 1321N1 cells (*P* = 0.04, uncorrected for multiple testing; Supplementary Material, Fig. S5B). We observed no difference when comparing individually (not in haplotypic combination) the SNP (Supplementary Material, Fig. S5C and D) and VNTR (Supplementary Material, Fig. S5E and F) alleles. There was no difference when the VNTR data were analysed as ‘short’ or ‘long’ according to the definition used by Arning *et al.* which reported association between this marker and degree of handedness (12) (Supplementary Material, Fig. S5G and H).

## Discussion

The aim of the present study was to understand the molecular mechanisms underlying the association with handedness at the *PCSK6* locus. We recently identified an intronic region of the *PCSK6* gene associated with relative hand skill through quantitative GWASs conducted in cohorts of children with dyslexia (5,6). An independent candidate gene study, which specifically targeted the *PCSK6* gene, found association between a VNTR at this same locus with degree of handedness in a cohort representative of the general population (12). Here we demonstrated experimentally that the associated locus contains a bidirectional promoter that regulates both a short sense *PCSK6* isoform and an antisense lncRNA. Furthermore, EMSA experiments showed that one of the top associated SNPs creates/disrupts the binding site for a nuclear factor(s) suggesting a direct causative effect on the bidirectional promoter.

Supported by independent studies, the *PCSK6* genetic association is statistically robust and is extremely interesting due to the well-established role of PCSK6 in activating NODAL to initiate patterning of the left/right body axis. While the associated locus is the same genomic region (Fig. 1), different markers were highlighted across the different studies. In particular, the candidate gene study conducted by Arning *et al.* implicated the rs10523972 VNTR as a possible functional variant (12). VNTR markers have been previously suggested to contribute to complex phenotypes, such as attention-deficit hyperactivity disorder (ADHD) (24). VNTRs can directly affect gene expression and therefore mediate genetic associations. VNTR alleles modify both the number and the spacing of transcription factors binding at a regulatory region (25). Because the VNTR was not genotyped in our original screenings (5,6), we reasoned that the GWAS results could be tagging a VNTR functional effect. We have now excluded this hypothesis and showed that rs11855415, one of the top associated SNPs in the original GWAS, has an allelic effect on DNA:protein binding affinity. We clearly demonstrate that the rs1185415 alleles alter the binding of a transcription factor(s) in a range of cell lines (Fig. 2).

While we cannot exclude functional effects of other markers within the same LD region, the role of rs11855415 is supported by different lines of evidence: (i) it was among the top associated markers (5,6), (ii) it is located close to a non-coding functional element and (iii) bioinformatics predictions returned the largest number of transcription factors potentially affected by allele-specific effects for this marker (Supplementary Material, Table S2). The other SNPs predicted to have allele-specific effects on DNA–protein binding affinity were rs1871978, rs9806256, rs4965830, rs7182874, rs752028 and rs882422 (Supplementary Material, Table S2). All these markers showed association with handedness [6; Supplementary Material, Fig. S1)] as expected because of the LD in the region. However, their allelic effects on DNA–protein biding affinity were predicted to have considerably less impact compared with rs11855415 (Supplementary Material, Table S2). Furthermore, none were located within predicted functional regions (Fig. 1; Supplementary Material, Fig. S1). Nevertheless, we tested rs7182874 which was the marker that showed the strongest association in the most recent GWAS (6). No allelic difference on DNA–protein binding affinity was observed (Fig. 2D).

Our results do not exclude that the VNTR might have an independent functional effect on the promoter activity. Therefore, we included VNTR alleles for comparison in dual-luciferase assays. However, we did not observe any strong allelic effect on promoter activity, neither for the rs11855415 SNP, the VNTR nor their haplotype combinations. While these results did not support an allelic effect on promoter activity they do not rule it out either. Intronic promoters, lncRNAs, enhancers and other non-coding functional elements are likely to have roles that are tightly regulated both temporally and spatially (26) and therefore putative allele-specific modulations might be detected only when all the relevant transcription machinery is in place. Interestingly, some *Hox* genes are predicted to be affected by rs11855415. *Hox* genes regulate embryonic anterior–posterior axis development at very specific time points and in very specific cells (27). In fact, the Nodal pathway itself, regulated by *PCSK6*, is active specifically during the first week of embryonic development. Therefore, it is possible that any genuine allele-specific effects might not be detectable through *in vitro* methodologies such as a luciferase assay, or might require specific cell types not used in this study.

We further investigated the functional elements of this region and confirmed, through a reporter assay, that this region acts as a bidirectional promoter. The activity was consistently stronger in the sense direction, as opposed to the antisense direction, across various cell lines (Fig. 3). In agreement with the ENCODE data (10), the strongest activity was observed in the K562 cells. We were able to detect both sense mRNA and antisense lncRNA transcripts that are expected to be regulated by the bidirectional promoter. While the antisense molecules could simply be a by-product of transcriptional activity, both transcripts have the potential to be functional. We demonstrated that the mRNA transcript starts with a novel exon and extends through to the *PCSK6* 3′ UTR. This transcript is predicted to be coding (Supplementary Material, Fig. S4). LncRNAs have been recognized to play various roles including the regulation of tissue-/time-specific gene expression patterns that contribute to complex processes such as neuronal differentiation and neurodevelopment (28). Bidirectional promoters may contribute to fine tuning the regulation of functional lncRNAs. Such mechanisms have been implicated in neurodevelopmental disorders and conditions, such as autism, fragile X syndrome and schizophrenia (29).

Therefore, it is possible that common variants affect the activity of this bidirectional promoter and, in turn, influence handedness-related phenotypes. Our genetic analysis does not support association with the VNTR, but does not exclude its independent effect. The inconsistent marker-traits associations across studies could be the result of differences in the study design. For example, we conducted our GWAS in a cohort consisting mainly of children selected for a clinical diagnosis of dyslexia. Handedness was assessed through the peg-board test, a task which gives a measure of how much an individual is more skilled at one task with one hand versus the other (5,6). Conversely, Arning *et al.* (12) screened a general population cohort of adults using the Edinburgh Inventory questionnaire, which measures the degree of handedness (how many tasks an individual performs preferentially with one hand versus the other) (11). Interestingly, the SNP associations replicated in three independent samples selected for dyslexia but not in a general population cohort (5,6), which is consistent with the lack of association reported for the rs1185415 by Arning *et al.* (12) in their general population sample. The sample analysed in the present study is derived from the one used in the initial GWAS (5) and was selected for a severe definition of dyslexia. While the SNP association replicated with same allelic trend in other two separate but less severe dyslexia samples, the association strength was attenuated (5), suggesting a dyslexia-specific effect. The dyslexia effect might also relate to properties of the phenotypes. PegQ distributions were very similar in the dyslexia and general population cohorts, but individuals with dyslexia tended to be slower with both hands at the peg-board test. However, the differences in marker-trait associations could depend on sampling effects in relatively small cohorts that inflate the association of one particular marker versus the other simply as a result of random fluctuations.

In summary, we have initiated the functional characterization of a locus associated with human handedness within *PCSK6*, a gene known to control the establishment of structural asymmetries. Our data suggest that the association between handedness and common variants is mediated by an intronic bidirectional promoter that controls both sense and antisense transcripts. These observations are in agreement with the increasing evidence that support the role of genetic variants within non-coding regions in influencing complex phenotypes through gene expression regulation (10). Our findings pave the way for future investigations aimed at understanding the function of the transcripts regulated by the bidirectional promoter in order to elucidate the mechanisms by which genes controlling structural laterality are also implicated in behavioural and functional asymmetries.

## Materials and Methods

### Genetic and bioinformatics analysis

We analysed the subset of the Oxford cohort that led initially to the discovery of the *PCSK6* association (*n* = 197 unrelated individuals) (5), which was selected on the basis of a severe dyslexia phenotype. The cohort was ascertained at the Royal Berkshire Hospital (Reading, UK) dyslexia clinic. This cohort has been described in detail previously (30–32). The subset analysed in this study was selected to represent the 200 probands with the most severe reading scores from the larger cohort. The initial ascertainment criteria included probands with a British abilities scales (BAS) single-word reading score >2 standard deviations (SDs) below that predicted by their intelligence quotient (IQ) derived from their verbal and non-verbal reasoning scores and at least one other sibling with a record of reading difficulties. These criteria included some probands with BAS scores within the ‘normal’ range because of high IQ scores. Therefore, for the remaining families, the criteria were changed such that the subject's difference in their BAS single-word reading score had to be ≥1 SD below the population mean for their age band, along with an IQ ≥90. The subgroup analysed here represent these different strategies with approximately two-thirds of the samples recruited under the first criteria.

Study participants performed the peg-board task (33), which involves moving a row of 10 pegs from one location to another with the left hand (L) and right hand (R) separately (7). From the average time for each hand on five trials, a measure of relative hand skill, PegQ [2(L − R)/(L + R)], was derived, as described previously (5,6).

Marker rs11855415, the top associated SNP in the original discovery sample, was imputed and then re-genotyped as part of a multiplex Sequenom assay (5). The VNTR rs10523972 alleles were determined by electrophoresis analysis of the PCR product as described by Arning *et al*. (12) to establish the number of repeats of the 33 bp sequence. Following standard quality controls procedures (34), quantitative association analysis was conducted using PLINK (35), modelling for an additive effect on a full association model; no evidence for population stratification was present in this sample. VNTR alleles were tested for association, both individually and pooled, as short (smaller than nine repeats) and long (nine or above repeats) consistent with the original report (12). LD analysis of the *PCSK6* region associated with PegQ was defined by visualising genotype data from the HapMap CEPH population (Rel28 PhaseII + III Aug10 NCBI36, dbSNP126) in Haploview v4.2 (36). All SNPs included in the LD region were queried for allelic effects on protein–DNA binding affinity using the TRANSFAC (17) and Genomatix MatInspector (18) databases. Promoter predictions were conducted with PROSCAN v1.7 (37) and the Mammalian Promoter Database (MPromDb) (38). Protein domain predictions were conducted with Interpro (39).

### Electrophoretic mobility shift assay (EMSA)

SH-SY5Y, HeLa, 1321N1 and K562 cells were cultured according to ECACC guidelines. Human Neural Stem Cells (hNSC) were cultured according to the provider's protocol (Life Technologies, Cat N7800-100). Isolation of nuclear protein extract was performed using the Signosis Nuclear Extraction kit (SK-0001, Signosis) according to the manufacturer's protocol. EMSAs were performed for 21 bp double-stranded complimentary biotinylated oligonucleotides containing each allele of the rs11855415 and rs7182874 SNPs (Eurofins MWG operon, see Supplementary Material for sequences). A detailed EMSA protocol is provided in Supplementary Material.

### Luciferase reporter assays

PCR products were generated from human DNA of individuals previously genotyped for both the rs11855415 SNP and the VNTR and presenting different haplotypic combinations. The PCR product sizes depended on the VNTR alleles: 1707 bp for the 6 alleles, 1806 bp for the 9 alleles and 1839 bp for the 10 alleles. Briefly, blunt-end products were cloned into the pCR™-Blunt II-TOPO vector (Invitrogen), for transformation and plasmids isolated. A double digest was performed between the *Kpn*I and *Xho*I restriction sites and then cloned in both sense and antisense directions (Fig. 1B) into the pGL4.10 luciferase vector (Promega). Site-directed mutagenesis using GeneArt Site-Directed Mutagenesis System (Invitrogen) was performed to synthesize the 6A and 10T constructs (which could not be derived from human genomic DNA) from the previously cloned 6T and 10A plasmids, respectively. The minimal promoter analysis was based on the analysis of progressively smaller fragments obtained by removing the sequence between the restriction sites for *Kpn*I (-1,171 bp from the TSS of the novel *PCSK6* isoform) and the sites for the following restriction enzymes: *Nde*l (−1067 bp), *Avr*II (−783 bp), *San*DI (−637 bp), *Stu*I (−507 bp), *PfI*MI (−378 bp), *AcI*I (−155 bp) and *Bsa*AI (+100 bp) from a fragment with the 10T VNTR/rs11855415 haplotype. All restriction enzymes were supplied by New England Biolabs (NEB). All constructs were verified by Sanger sequencing using the service provided by the MRC PPU DNA Sequencing and Services, at the University of Dundee.

Allelic differences on promoter activity were tested using the Dual-Glo Luciferase System (Promega). All luciferase reporter constructs were assayed 24 h after transfection. All experiments were repeated at least three times in triplicate.

Statistical tests were performed using SPSS for Windows version 21. For the luciferase assay, the promoter activity of each construct was normalized to a thymidine kinase Renilla plasmid (pRL-TK) and the reporter gene activity expressed as fold change (FC) over the empty pGL4 vector. Tests for significant differences in luciferase activity between the rs11855415 alleles were performed using Student's paired two-tailed *t*-test. VNTR alleles were compared by single-factor analysis of variance (ANOVA) test.

### Transcript detection

Total RNA was extracted from each cell line using the RNeasy Mini kit (Qiagen). Five micrograms of DNase-treated RNA (Ambion DNA-free Kit, Invitrogen) was used for cDNA synthesis using oligo-d(T) primers as part of the Superscript III assay (Invitrogen) and according to the manufacturer's protocol. PCR fragments were gel extracted (QIAquick Gel Extraction Kit, Qiagen) and sequenced by Sanger sequencing. All primers were designed using Primer3 (40) and are listed in Supplementary Material, Table S3.

## New sequence accession numbers (EMBL)

*PCSK6-AS1* (HGNC ID: 51448, EMBL Accession Nos LN713949-LN713951), *PCSK6-AS2* (HGNC ID: 51448, EMBL Accession No LN713952), Novel *PCSK6* isoform (EMBL Accession No LN714797).

## Supplementary Material

Supplementary Material is available at *HMG* online.

## Funding

S.P. is a Royal Society University Research Fellow. This work was supported by the Royal Society (grant number RG110387 to S.P.); R.S. was supported by a University of St Andrews PhD scholarship. Genotyping at the Wellcome Trust Centre for Human Genetics was supported by the Wellcome Trust (090532/Z/09/Z) and a Medical Research Council Hub grant (G0900747 91070). Genetic analysis was supported by the St Andrews Bioinformatics Unit funded by the Wellcome Trust (097831/Z/11/Z). D.F.N. is an MRC Career Development Fellow supported by the MRC (G1000569/1 and MR/J003719/1). Funding to pay the Open Access publication charges for this article was provided by the Wellcome Trust.

## Supplementary Material

Supplementary Data
