# Supplementary material for: The handedness-associated PCSK6 locus spans an intronic promoter regulating novel transcripts
Source: Hum Mol Genet. 2016 Feb 21;25(9):1771–9. doi: 10.1093/hmg/ddw047 (PMC4986331; doi:10.1093/hmg/ddw047)
Supplement: Supplementary Data [file supp_25_9_1771__index.html]

The handedness-associated PCSK6 locus spans an intronic promoter regulating novel transcripts — The handedness-associated PCSK6 locus spans an intronic promoter regulating novel transcripts — The handedness-associated PCSK6 locus spans an intronic promoter regulating novel transcripts — Supplementary Data 

# The handedness-associated *PCSK6* locus spans an intronic promoter regulating novel transcripts

## Supplementary Data

Supplementary Data

- Supplementary Data - Docx file
