# Supplementary material for: The handedness-associated PCSK6 locus spans an intronic promoter regulating novel transcripts
Source: Hum Mol Genet. 2016 Feb 21;25(9):1771–9. doi: 10.1093/hmg/ddw047 (PMC4986331; doi:10.1093/hmg/ddw047)
Supplement: Supplementary Data [file supp_ddw047_ddw047supp.docx]

**Supplementary Material**

*EMSA Protocol*

Double-stranded oligonucleotide probes in 20 fmol, 200 fmol and 2 pmol aliquots were prepared by annealing 5’-biotin labelled oligonucleotides in annealing buffer (10 mM Tris pH 7.5-8, 1 mM EDTA, 50 mM NaCl) overnight to room temperature after incubation at 95 °C for 2 minutes. Protein concentrations were determined using a Qubit Protein Assay (Invitrogen). A binding reaction of nuclear protein extract (7.5 µg), 0.5 µg poly d(I-C) (Sigma Aldrich, UK) and 3 μl 5X Binding Buffer (1 mL 1 M Hepes pH 8.0, 2.5 mL 1 M KCl, 25 μl 1 M DTT, 5 μl 0.5 M EDTA, 50 μl 1 M MgCl2, 2.5mL glycerol dH2O to 10 mL) was incubated with labelled probe (20 fmol), with 10x/100× unlabelled competitors or scrambled oligos when necessary, at 25 °C for 20 min. Following incubation, samples were separated by electrophoresis with non-denaturing 5% polyacrylamide minigels (40% acrylamide/bisacrylamide (29:1)). The gels were then electroblotted to a Nylon B positive membrane (Thermo Scientific, UK) for 45 minutes at 100 V in 4°C 0.5x TBE buffer. The protein-DNA complexes were autocross-linked to the nylon membrane with a Stratalinker 1800 UV transilluminator using 312 nm bulbs. Blocking of the membrane and subsequent chemiluminescent detection of the biotin-labelled DNA was performed using an enhanced luminol substrate for horseradish peroxidase (HRP) (Thermo Scientific, USA) and visualised using a Fuji LAS-3000 imaging system.

Probes for the rs11855415 SNP were generated by incubating the oligonucleotide containing the T or A allele ( 5’-AGTCCAATAA[A/T]AATGAAGTCT) with the corresponding biotinylated oligos ( 5’-BIO-AGACTTCATT[A/T]TTATTGGACT) or unlabelled oligos (5’-AGACTTCATT[A/T]TTATTGGACT) to generate cold competitors. The sequence of the scrambled probe was 5’-GCCTGTCACCCGTCATGTAT. Probes for the rs7182874 SNP were generated by incubating biotinylated oligos containing the C or T alleles (5’-BIO-AAGCTGGCCC[C/T]GCTGGAAGG) with unlabelled oligos (5’- CCTTCCAGC[A/G]GGGCCAGCTT). Cold competitors were generated on combining AAGCTGGCCC[T/C]GCTGGAAGG and 5’- CCTTCCAGC[A/G]GGGCCAGCTT oligos.

**Supplementary figures**

**
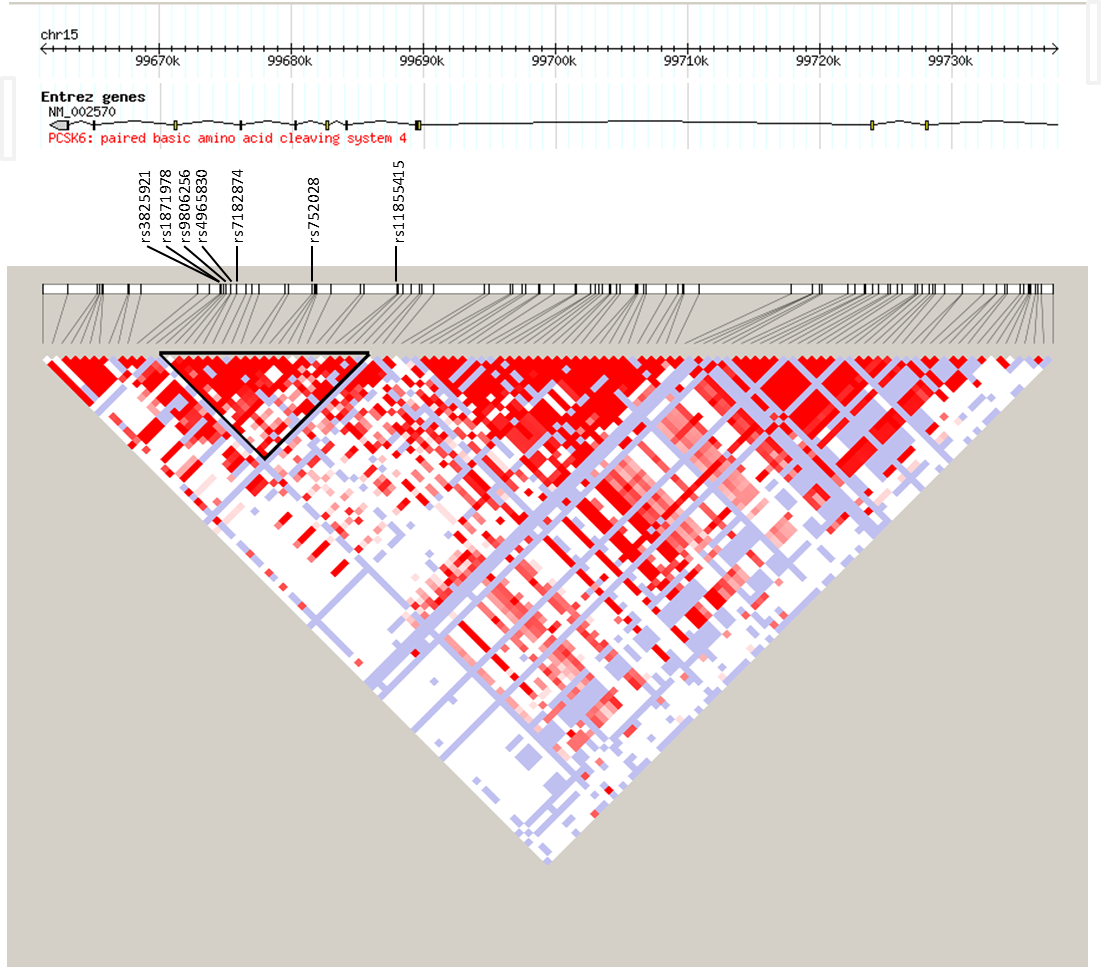
**

**Figure S1** LD structure across the PCSK6 locus generated from HapMap European population (CEPH) visualised with Haploview v4.2. Top track indicates the chromosomal region and the corresponding portion of the PCSK6 gene (exons 22 to 12 from left to right). Top associated SNPs (P < 1 x 10^-5^) in previous GWASs for handedness (1, 2) cluster in a region marked by a black triangle (see also Figure 1). A red box indicates the absolute D prime (D’) between two loci while an empty or blue box represents low LD.

**
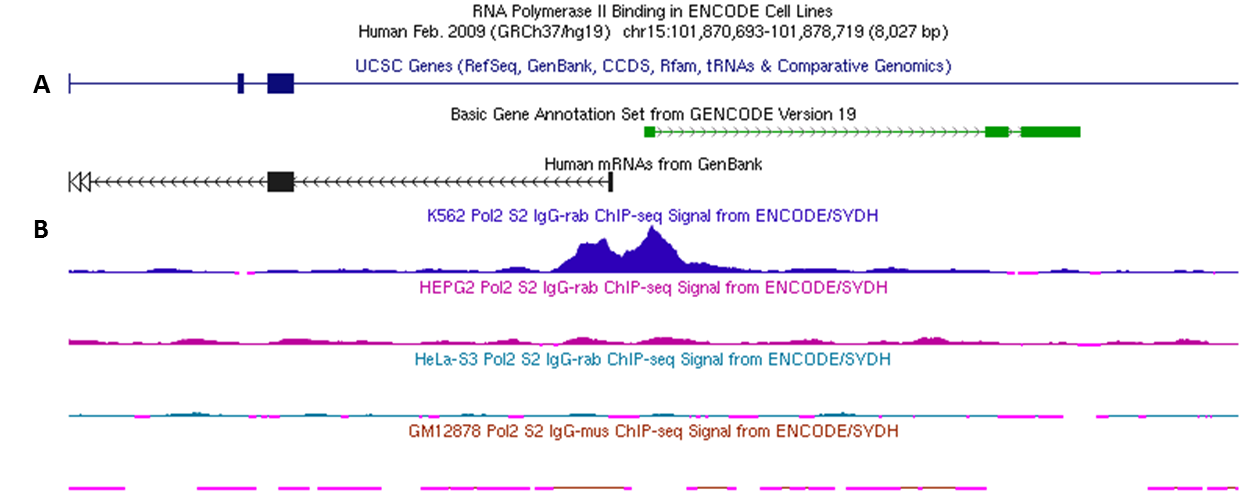
**

Figure S2 RNA Polymerase II binding in ENCODE Cell Lines UCSC Genome Browser track (A) displays the PCSK6 gene locus (blue) with sense (black) and antisense (green) transcripts originating from the locus (B) Chip-SEQ data indicates RNA Polymerase II binding in the K562 cell line (blue) to be substantially higher compared to other cell lines HEPG2 (purple), HeLa (turquoise) and GM12878 (pink).

**
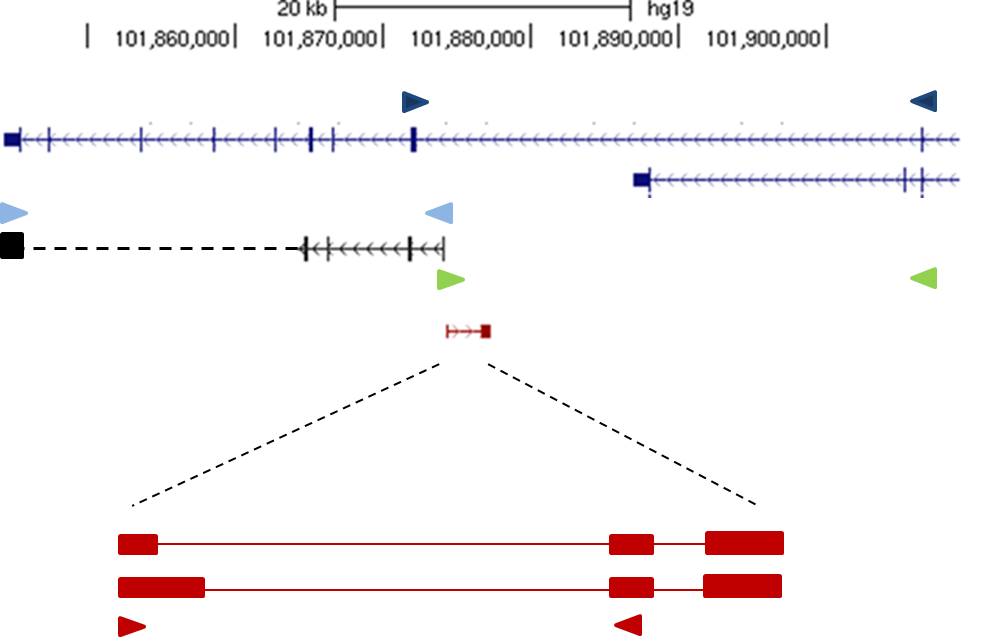
**

**A**

**
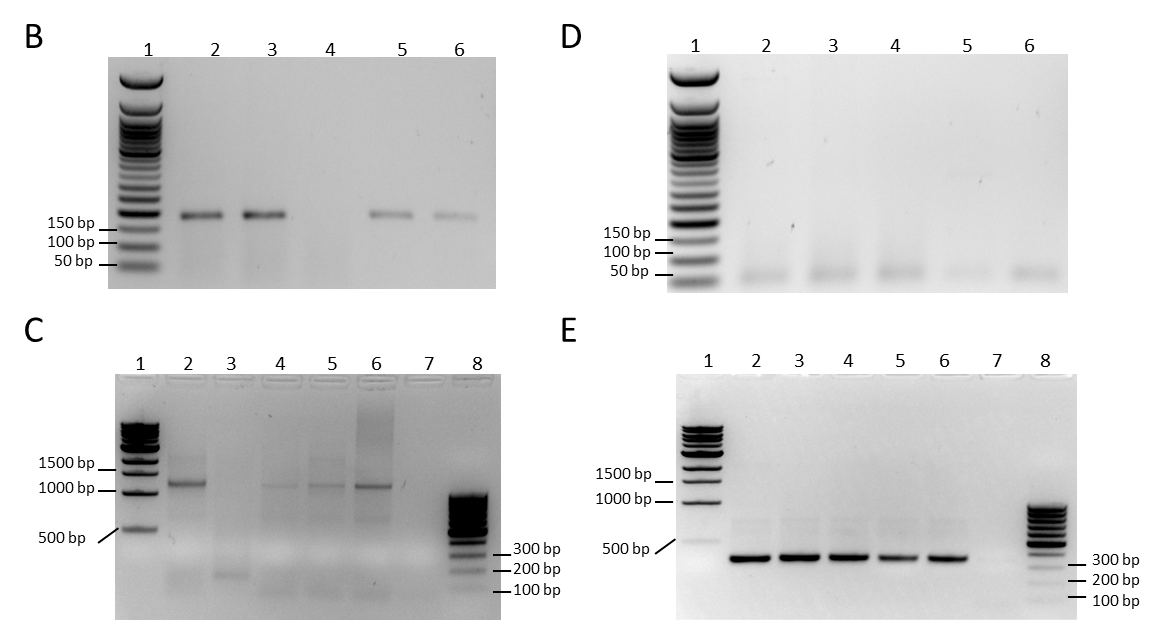
**

**Figure S3 PCSK6 transcripts regulated by the promoter at the handedness associated locus. A)** Location of primers used to detect the different transcripts (see also Figure 1). Dark blue triangles represent primers targeting exons 13 and 14 of the full length *PCSK6* transcript aimed at testing for alternative spliced isoforms including the novel exon. Light blue triangles represent primers targeting the first novel exon of the short *PCSK6* isoform and the 3’ UTR of the full length *PCSK6*. The dotted line of this transcript represents the sequence that extends beyond what predicted by the spliced EST and GENCODE tracks from the UCSC Genome Browser (<http://genome-euro.ucsc.edu>). Green triangles represent primers designed to test whether the novel exon is present within other transcripts. Red triangles represent primers targeting exons 1 and 2 of the antisense lncRNA (zoomed out visualization at the bottom of panel A). **B)** PCR products, obtained using the dark blue primers, show a single band of 211 bp. No alternative splicing and no inclusion of additional exons between exon 13 and 14 of the full length *PCSK6* is observed. **C)** PCR products of 1247 bp obtained using the light blue primers and confirming a *PCSK6* transcript that spans from the novel exon through the 3’ UTR (shown also in Figure 3). **D)** No amplification is observed when using the green primers confirming the novel exon is not included with exons upstream of the bidirectional promoter. **E)** Amplification of β-actin transcript (353 bp), a house-keeping gene, demonstrating the quality of the cDNA used in B), D) and C). Cell line order from left to right in B), D) C) and E) is K562, HeLa, 1321N1, hNSC, SH-SY5Y. Reference ladders are 1 kb and 100 bp in C) and E), and 50 bp in B) and D).

**A**


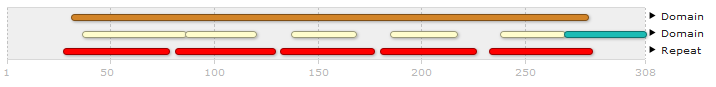


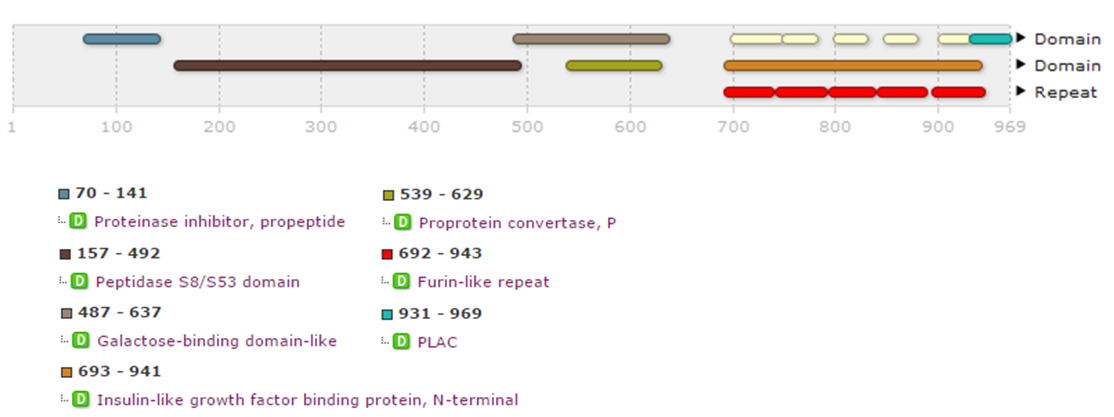


**B**

**Figure S4** *PCSK6* protein domain predictions. **A)** Prediction for the *PCSK6* short isoform regulated by the bidirectional promoter compared to the **B)** full length *PCSK6.* The short isoform is predicted to code for the same domains at the C-terminus of the full length *PCSK6*. The short isoform does not include the convertase domain. Predictions were conducted with InterProScan (3).

**
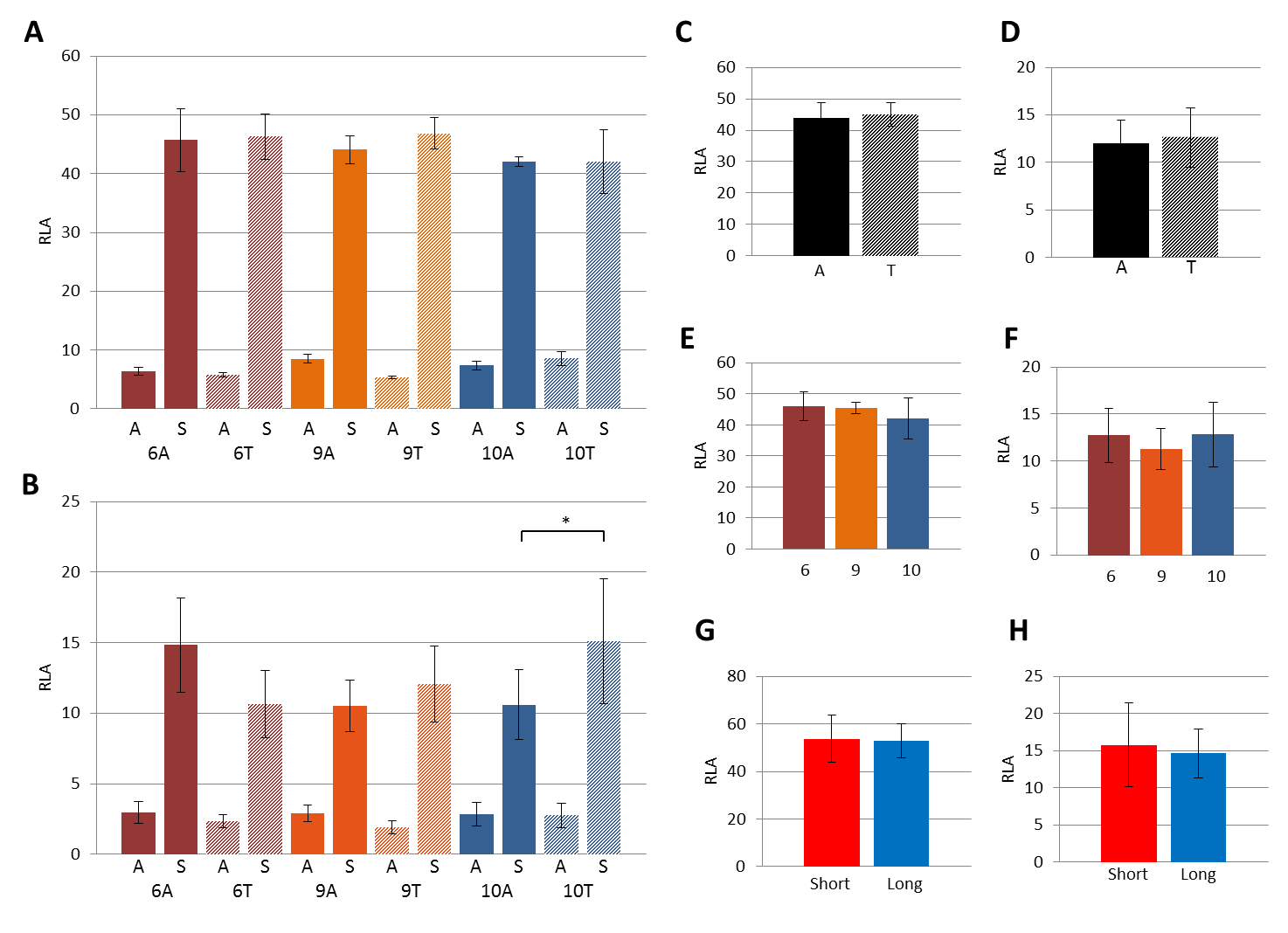
**

**Figure S5.** Dual luciferase assay results testing for allelic effects on promoter activity. Luciferase assay were conducted in K562 **(A, C, E** and **G)** and 1321N1 (**B, D, F** and **H**) cell lines. The alleles at rs1185415 (A as solid bars; T as striped bars) and at the VNTR (6 is red; 9 is orange; and 10 is blue) were compared in different haplotypic combinations both in the antisense (A) and sense (S) direction (A and B). The rs11855415 alleles were compared regardless of the VNTR background (**C** and **D**). The VNTR alleles were analysed individually (**E** and **F**) or as short (6 repeats in bright red) and long (9 and 10 repeats in light blue; **G** and **H**), regardless of the SNP background. Luciferase expression was measured relative to the empty pGL4 vector following renilla normalisation. Data are representative of at least 3 independent experiments performed in triplicate and are expressed as Mean±SD of normalized luciferase activity (n = 3); RLA = Relative Luciferase Activity; * indicates P-value of less than 0.05.

**Supplementary Tables**

| **Table S1. Association analysis results across different studies** | | | | | | | |  |  |  |  |  |  |  |  |  |
| --- | --- | --- | --- | --- | --- | --- | --- | --- | --- | --- | --- | --- | --- | --- | --- | --- |
|  | **Scerri et al(2)** | | | | | | | | **Arning et al(4)** | | | | **Present study** | | | |
|  | Discovery dyslexia sample | | | | Total (dyslexia) | | | |  | | | | Subset of discovery sample | | | |
| **Marker** | PegQ | | | | | | | | Handedness direction | | | | PegQ | | | |
|  | N | P | β | SE | N | P | β | SE | N | P | β | SE | N | P | β | SE |
| **rs11855415 SNP** | 191 | 4.7 x 10^-7^ | 0.6 | 0.12 | 744 | 1.99x10^-8^ | 0.4 | NA^a^ | 1113 | 0.84 | NA | NA | 162 | 4.8 x 10^-4^ | 0.03 | 0.001 |
| **rs10523972 VNTR^b^** | NA | NA | NA | NA | NA | NA | NA | NA | 1113 | 0.001 | NA | NA | 162 (188) | 0.61 (0.61) | -0.005 (-0.004) | 0.01 (0.009) |
| ^a^ Calculated as 95% confidence interval for β as 0.23-0.47 | | | | | | |  |  |  |  |  |  |  |  |  |  |
| ^b^ Result presented only for short/long allele definition | | | | | | |  |  |  |  |  |  |  |  |  |  |
| Abbreviations: NA=not available; SE = standard error; β = the mean effect size of each copy of the minor allele measured in standard deviation | | | | | | | | | | | | | | | | |
|  |  |  |  |  |  |  |  |  |  |  |  |  |  |  |  |  |

Table S2 Allelic effects of common variants at the PCSK6 locus on transcription factor binding affinity

| **SNP** | **Allele** | **Transcription Factors** |
| --- | --- | --- |
| rs3825921 | A | None |
| rs3825921 | G | None |
| rs1871975 | C | None |
| rs1871975 | T | None |
| rs1871976 | A | None |
| rs1871976 | G | None |
| rs1871978 | C | HSF 1  NF-E2-related factor 1/Transcription Factor MafG heterodimers binding to subclass of AP1 sites |
| rs1871978 | T | MyT1  RREB-1 |
| rs9806218 | A | None |
| rs9806218 | G | None |
| rs9806256 | C | Cardiotrophin-1 |
| rs9806256 | T | Nuclear transcription factor Y  Binding site for a Pbx1/Meis1 heterodimer |
| rs4965830 | A | Homeo box C10/Hox-3iota  Muscle TATA box |
| rs4965830 | T | Intestine specific homeodomain factor for Homeobox protein CDX-1  Homeobox protein Hox-B9  Homeobox protein Hox-C13  Homeobox protein Hox-D12 |
| rs2220055 | A | None |
| rs2220055 | G | None |
| rs2277593 | C | None |
| rs2277593 | G | None |
| rs2277593 | T | None |
| rs7182874 | C | Paired box protein Pax-5  Protein BANP  Krueppel-like factor 6 |
| rs7182874 | T | T-cell acute lymphocytic leukemia protein 1 |
| rs12901236 | C | Inhibitor of growth protein 4 |
| rs12901236 | T | Inhibitor of growth protein 4 |
| rs1471656 | C | None |
| rs1471656 | T | None |
| rs1947942 | A | Xvent-1 protein |
| rs1947942 | G | None |
| rs752028 | C | None |
| rs752028 | T | Breast cancer type 1 susceptibility protein: Upstream stimulatory factor 2 complex |
| rs882422 | A | Krueppel-like factor 6 |
| rs882422 | G | Krueppel-like factor 6  Transcription intermediary factor 1-beta  BEN domain-containing protein |
| rs752026 | A | None |
| rs752026 | G | None |
| rs755867 | A | None |
| rs755867 | G | None |
| rs2073592 | C | None |
| rs2073592 | T | None |
| rs2239858 | A | Killer cell lectin-like receptor subfamily G member 1 |
| rs2239858 | G | Killer cell lectin-like receptor subfamily G member 1 |
| rs12916087 | A | None |
| rs12916087 | G | None |
| rs12900794 | C | None |
| rs12900794 | T | None |
| rs11855415 | A | Sex-determining region Y protein  POU domain, class 6, transcription factor 1  Homeobox protein Hox-A5  POU domain, class 4, transcription factor 3  Homeobox protein BarH-like 2  POU domain, class 2, transcription factor 1  Homeobox protein Hox-B3  LIM-homeodomain transcription factor  Homeobox protein Nkx-6.3  DNA-binding protein SATB1  GS homeobox 1  Zinc finger protein 333 |
| rs11855415 | T | POU domain, class 3, transcription factor 2  Intestine specific homeodomain factor for Homeobox protein CDX-1  Homeobox B8 / Hox-2delta  Spalt-like transcription factor 1  Special AT-rich sequence-binding protein 1, predominantly expressed in thymocytes, binds to matrix attachment regions (MARs)  Homeobox D10 |

Predictions were obtained by querying the TRANSFAC (v2014.4)(5) and Matinspector (MatInspector Release 8.0.6)(6) databases

Table S3 List of PCR primers used in this study

| **Fragment** | **Sequence** | **Size (bp)** | **Primer in**  **FigS3** |
| --- | --- | --- | --- |
| PCSK6 antisense | F: GGTGCAGAAAACAAGCCTG  R: CTTCCCTGCTGGCGTTTTTG | 112;232* | Red |
| PCSK6 short isoform | F: GAACAACTTCCTGTGTCACTGC  R: ATGCTGCTCCTGGGGAGATA | 1247 | Light blue |
| PCSK6 Exon 13-14 | F: ATGCTGCTCCTGGGGAGATA  R: CTGATGGGCACTGAAGGTGT | 211 | Dark blue |
| Novel exon - Exon 13 | F: CGCTGCAGCAGTGACACAGGA  R: ATGCTGCTCCTGGGGAGATA | 300 | Green |
| B-Actin control | F: GCTCGTCGTCGACAACGGCTC  R: CAAACATGATCTGGGTCATCTTCTC | 353 |  |
| Luciferase construct | F: CTGGCTCTAAATGGCAGCCT  R: ACCCCGAGTACTACTGCTTTT | 1707-1839 |  |
| mutagenesis  6T->6A  10A->10T | F: AGAGAGACTTCATTATTATTACACTCTCT  R: AGAGAGTGTAATAATAATGAAGTCTCTCT  5’-AGAGAGACTTCATTTTTATTACACTCTCT  5’-AGAGAGTGTAATAAAAATGAAGTCTCTCT |  |  |

* Indicates PCSK6as_V2 isoform size

**References**

1 Brandler, W.M., Morris, A.P., Evans, D.M., Scerri, T.S., Kemp, J.P., Timpson, N.J., St Pourcain, B., Smith, G.D., Ring, S.M., Stein, J. *et al.* (2013) Common variants in left/right asymmetry genes and pathways are associated with relative hand skill. *PLoS Genet.*, **9**, e1003751.

2 Scerri, T.S., Brandler, W.M., Paracchini, S., Morris, A.P., Ring, S.M., Richardson, A.J., Talcott, J.B., Stein, J. and Monaco, A.P. (2011) PCSK6 is associated with handedness in individuals with dyslexia. *Hum. Mo.l Genet.*, **20**, 608-614.

3 Mitchell, A., Chang, H.Y., Daugherty, L., Fraser, M., Hunter, S., Lopez, R., McAnulla, C., McMenamin, C., Nuka, G., Pesseat, S. *et al.* (2015) The InterPro protein families database: the classification resource after 15 years. *Nucleic Acids Res.*, **43**, D213-221.

4 Arning, L., Ocklenburg, S., Schulz, S., Ness, V., Gerding, W.M., Hengstler, J.G., Falkenstein, M., Epplen, J.T., Gunturkun, O. and Beste, C. (2013) VNTR Polymorphism Is Associated with Degree of Handedness but Not Direction of Handedness. *PLoS One*, **8**, e67251.

5 Matys, V., Kel-Margoulis, O.V., Fricke, E., Liebich, I., Land, S., Barre-Dirrie, A., Reuter, I., Chekmenev, D., Krull, M., Hornischer, K. *et al.* (2006) TRANSFAC and its module TRANSCompel: transcriptional gene regulation in eukaryotes. *Nucleic Acids Res.*, **34**, D108-110.

6 Cartharius, K., Frech, K., Grote, K., Klocke, B., Haltmeier, M., Klingenhoff, A., Frisch, M., Bayerlein, M. and Werner, T. (2005) MatInspector and beyond: promoter analysis based on transcription factor binding sites. *Bioinformatics*, **21**, 2933-2942.
